# Supplementary material for: Estimating the contribution of musculoskeletal impairments to altered gait kinematics in children with cerebral palsy using predictive simulations
Source: J Neuroeng Rehabil. 2025 Oct 28;22:225. doi: 10.1186/s12984-025-01767-w (PMC12570557; doi:10.1186/s12984-025-01767-w)
Supplement: Supplementary file 1 — Supplementary Material 1. [file 12984_2025_1767_MOESM1_ESM.docx]

**Supplementary material: Estimating the contribution of musculoskeletal impairments to altered gait kinematics in children with cerebral palsy using predictive simulations**

*Bram Van Den Bosch^1^, Lars D’Hondt^1^, Ilse Jonkers^1^, Kaat Desloovere^2,3^, Anja Van Campenhout^4,5^, Friedl De Groote^1^*

^1^Department of Movement Sciences, KU Leuven, Leuven, Belgium

^2^Department of Rehabilitation Sciences, KU Leuven, Leuven, Belgium

^3^Clinical Motion Analysis Laboratory, University Hospitals Leuven, Pellenberg, Belgium

^4^Department of Development and Regeneration, KU Leuven, Leuven, Belgium

^5^Department of Orthopedics, University Hospitals Leuven, Leuven, Belgium

Contents

[1. Model scaling 2](#_Toc207640689)

[2. Manual Muscle Testing 3](#_Toc207640690)

[3. Marker protocol 4](#_Toc207640691)

[4. Post hoc analysis of selected scaling factors based on Manual Muscle Testing 6](#_Toc207640692)

[5. Scaling factors for modeling contractures and weakness 8](#_Toc207640693)

[6. Sensitivity analysis contractures 9](#_Toc207640694)

[7. Moment arm deficits 11](#_Toc207640695)

[8. Motor control clinical examination data 13](#_Toc207640696)

[9. Shapley values 14](#_Toc207640697)

[10. SPM1D analysis 16](#_Toc207640698)

[11. Simulated and experimental kinematics per subject 18](#_Toc207640699)

[12. RMSD and CC differences between models, per subject 22](#_Toc207640700)

[13. References 26](#_Toc207640701)

1. Model scaling

In all models, the radii ($r_{CS}$), stiffness ($k_{CS}$), and damping ($d_{CS}$) of the contact spheres were scaled to account for anthropometric differences (length and mass) between participants:

$r_{CS, subject}=r_{CS, DHondt{2024}_{3seg}}*\left( \frac{L_{subject}^{foot}}{L_{DHondt{2024}_{3seg}}^{foot}} \right)$ , (S1)

$k_{CS,subject}=k_{CS, DHondt{2024}_{3seg}}*\left( \frac{M_{subject}}{M_{DHondt{2024}_{3seg}}}* \left( \frac{L_{DHondt{2024}_{3seg}}}{L_{subject}} \right)^{2} \right)$ , (S2)

$d_{CS,subject}=d_{CS,DHondt{2024}_{3seg}}*\left( \frac{L_{subject}}{L_{DHondt{2024}_{3seg}}} \right)$ , (S3)

were $L^{foot}$ refers to the length of the foot, $L$ refers to body height and $M$ refers to body weight.

Stiffness and damping of all DOFs as well as stiffness and damping coefficients in the the terms in the coordinate limit torques were scaled with $s_{j}$:

$s_{j}= \frac{M_{subject} * L_{subject}}{M_{DHondt2024}* L_{DHondt2024}}$, (S4)

with $M_{subject}$ the mass of the subject, $L_{subject}$ the height of the subject, and $M_{DHondt{2024}}$ and $L_{DHondt{2024}}$ the mass and height of the generic model.

1. Manual Muscle Testing

The strength is evaluated for the full active range of motion (ROM) as well as in a predefined position. When the active range of motion is smaller than the passive range of motion (for instance due to co-contraction of the antagonists), the strength-score is not reduced, but the limits in active range of motion are noted, and the score for selectivity is reduced.

**Table S1**

*Manual Muscle Testing scoring system*

| **Score** | **Description** |
| --- | --- |
| 0 | Contraction cannot be palpated |
| 1 | Evidence of slight contraction of the muscle, but joint motion is not visible |
| 2 | Complete ROM in gravity eliminated plane (ROM can be slightly decreased because of co-contraction) |
| 3 | Perfect motion against gravity (almost full available ROM, ROM can be slightly decreased because of co-contraction) |
| 4 | Motion against gravity with some (moderate) resistance (full available ROM) |
| 5 | Motion against gravity with maximal resistance (full available ROM) |

In children with CP a lack of control of pelvis and trunk motion is frequently observed. Therefore, a specific evaluation of abdominal and back muscles is also performed.

**Table S2**

*Manual Muscle Testing scoring system for abdominal muscles*

| **Score** | **Description** |
| --- | --- |
| 0 | No visible / palpable abdominal contraction |
| 1 | Palpable contraction, but cannot elicit cervical and trunk flexion |
| 2 | Can raise head off the mat into cervical flexion |
| 3 | Can lift shoulders and scapulae off the mat |
| 4 | Can perform thoracic flexion |
| 5 | Can sit up with trunk flexion |

**Table S3**

*Manual Muscle Testing scoring system for back muscles*

| **Score** | **Description** |
| --- | --- |
| 0 | Cannot attempt any movement and no contraction can be palpated |
| 1 | Palpable contraction as the patient performs cervical extension (to raise head off the mat) |
| 2 | Can raise head and shoulders off the mat |
| 3 | Can raise chest and ribs off the mat |
| 4 | Can perform lumbar extension |
| 5 | Can perform lumbar extension with hip extension |

1. Marker protocol

**Table S4**

*Marker protocol*

| **Segment** | **Marker ID** | **Anatomical reference position** | **Details** | **3D movement analyses** | **Glycerin markers MRI** |
| --- | --- | --- | --- | --- | --- |
| **Torso** | LSHO  RSHO | Left/Right acromion | Most proximal point. | x |  |
|  | CLAV | Upper edge of sternum/Jugular notch | In the middle. | x |  |
|  | STRN | Lower edge of sternum/Xiphoid process | M In the middle. | x |  |
|  | C7 | 7^th^ Cervical vertebrae | Spinous process.  In the middle. | x |  |
|  | T10 | 10^th^ Thoracic vertebrae | Spinous process.  In the middle. | x |  |
| **Pelvis** | LASI  RASI | Left/Right anterior superior iliac spine | Most pronounced part. | x | x |
|  | LPSI  RPSI | Left/Right posterior superior iliac spine | Below the dimple (if visible) or most pronounced part. | x | x |
|  | LLAT  RLAT | Left/Right lateral midpoint | Highest point of the iliac crest. | x | x |
| **Thigh** | LTHI  RTHI | Wand, Lower 1/3 of thigh (lateral side) |  | x |  |
|  | LF1  RF1 | Cluster marker thigh  Downward | Exact placement not critical. | x | x |
|  | LF2  RF2 | Cluster marker thigh  Dorsal | Exact placement not critical. | x | x |
|  | LF3  RF3 | Cluster marker thigh  Ventral | Exact placement not critical. | x | x |
|  | LKNE  RKNE | Lateral epicondyle of knee | Most pronounced part of epicondyle. Palpate from proximal with knee straight. Markers should hardly move while bending the knee. | x | x |
|  | LMEK  RMEK | Medial epicondyle of knee | Most pronounced part of epicondyle. Palpate from proximal with knee straight. Markers should hardly move while bending the knee | x | x |
| **Shank** | LTIB  RTIB | Wand, lower 1/3 of shank (anterior side) |  | x |  |
|  | LT1  RT1 | Cluster marker thigh Downward | Exact placement not critical. | x | x |
|  | LT2  RT2 | Cluster marker thigh  Dorsal | Exact placement not critical. | x | x |
|  | LT3  RT3 | Cluster marker thigh  Ventral | Exact placement not critical. | x | x |
| **Ankle** | LLANK  RLANK | Lateral malleolus | Most pronounced part. | x | x |
|  | LMANK  RMANK | Medial malleolus | Most pronounced part. | x | x |
|  | LSTL  RSTL | Sustaniculum Tali | Medial aspect of calcaneus, equidistant from HEE | x | x |
|  | LLCA  RLCA | Lateral calcaneus | Lateral aspect of calcaneus, equidistant from HEE | x | x |
| **Foot** | LHEE  RHEE | Calcaneus (dorsal part) | At same height as TOE, with the foot flat on the ground. | x | x |
|  | LTOE  RTOE | 2^nd^ Metatarsal head | Over the 2th metatarsal head on the midfoot side of the equines break between for- and midfoot. | x | x |
|  | LCM5  RCM5 | 5^th^ Metatarsal head | On top of 5^th^ metatarsal head. | x | x |
| Total amount of markers functional movements: 34 + 4 clusters of 3 = **46** | | | | | |
| Total amount of markers MRI: **36** | | | | | |

1. Post hoc analysis of selected scaling factors based on Manual Muscle Testing

We performed a post hoc analysis in which we correlated scaling factors obtained from MMT with scaling factors derived from an instrumented strength assessment [1] in a different group of 34 children with CP (19 boys; mean age 8.8 ± 2.2 years; body mass 29 ± 9 kg; height 1.29 ± 0.14 m) for whom both measures were available (54 measured legs, unpublished data). Note that only strength of the knee flexors and extensors, and ankle plantar flexors was evaluated with the instrumented strength assessment. Scaling factors derived from the instrumented strength assessment were the ratio of the joint torque of the child and the median joint torque of typically developing children of the same dimensions (body mass x height) during the instrumented assessment [2]. We found that there is a large variability in MMT scores where the same score corresponded to a wide range of dynamometer-based scaling factors, but that the least squares fit of dynamometer-based versus MMT-based corresponds best with the identity line for the set of scaling factors chosen for the simulations in this study (Fig. S1).

*
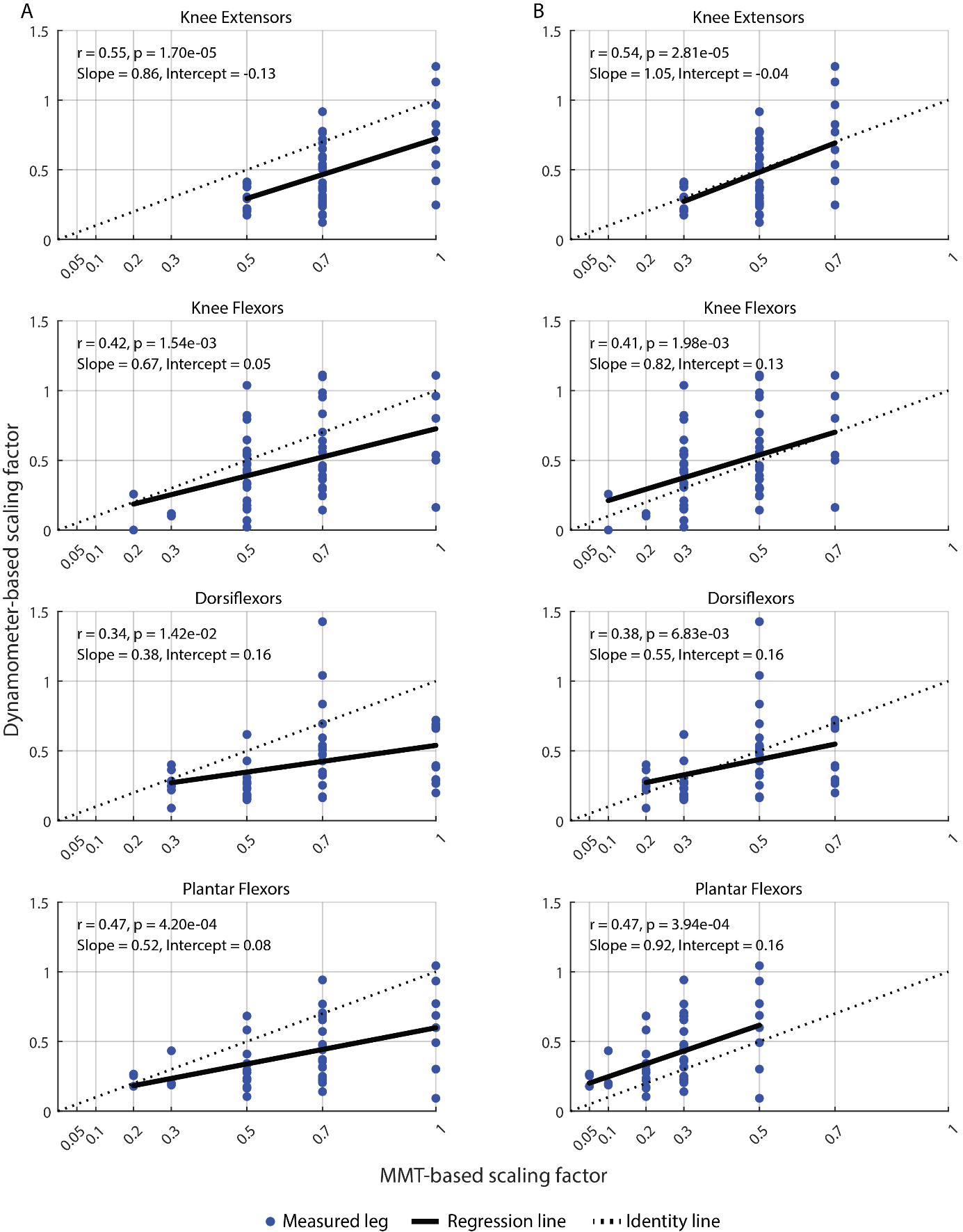
*

*Fig. S1: Dynamometer- and MMT-based strength scaling factors.* *The dynamometer-based scaling factor is the ratio between the measured (dynamometer) torque of the child and the median torque of a typically developed child with the same dimensions (body mass x height)* [2]*. The MMT-based scaling factor is the scaling factor we associated with the MMT score measured during the clinical exam. A) MMT-based scaling factors of 1.0, 0.7, 0.5, 0.3, 0.2 and 0.1 for MMT scores of 5, 4, 3, 2, 1, 0, respectively. B) MMT-based scaling factors of 0.7, 0.5, 0.3, 0.2, 0.1, and 0.05 for MMT scores of 5, 4, 3, 2, 1, 0, respectively. For plantar flexors we reduced the scaling factor by one step (e.g. a score of 3 corresponds to a scaling factor of 0.2 instead of 0.3).*

1. Scaling factors for modeling contractures and weakness

**Table S5**

*Scaling factors for modeling contractures and weakness*

|  | CP1 | | | CP2 | | CP3 | | | | CP4 | | CP5 | | | CP6 | | | CP7 | | | | CP8 | | |
| --- | --- | --- | --- | --- | --- | --- | --- | --- | --- | --- | --- | --- | --- | --- | --- | --- | --- | --- | --- | --- | --- | --- | --- | --- |
|  | L | R | L | | R | | L | R | L | | R | | L | R | | L | R | | L | R | L | | R |  |
| Optimal fiber length (GEN) |  |  |  | |  | |  |  |  | |  | |  |  | |  |  | |  |  |  | |  |  |
| *Soleus* | - | - | 0.81 | | 0.67 | | 0.78 | 0.65 | 0.69 | | - | | - | - | | - | - | | 0.58 | 0.75 | - | | - |  |
| *Gastrocs* | - | - | - | | 0.90 | | 1.03 | - | 0.93 | | - | | - | - | | - | - | | 0.76 | 0.82 | - | | - |  |
| *Hamstrings* | 0.76 | 0.78 | 0.82 | | 0.83 | | 0.94 | 0.92 | 0.73 | | 0.83 | | 0.76 | 0.84 | | 0.81 | 0.76 | | 0.88 | 0.90 | 0.83 | | 0.80 |  |
| *Rectus femoris* | 0.80 | 0.80 | - | | 0.90 | | - | 0.90 | - | | 0.80 | | - | - | | - | - | | 0.90 | 0.90 | - | | - |  |
| *Iliopsoas* | - | - | - | | - | | - | - | 0.65 | | 0.90 | | - | - | | 0.78 | - | | 0.90 | 0.92 | 0.75 | | 0.76 |  |
|  |  |  |  | |  | |  |  |  | |  | |  |  | |  |  | |  |  |  | |  |  |
| Optimal fiber length (GEO) |  |  |  | |  | |  |  |  | |  | |  |  | |  |  | |  |  |  | |  |  |
| *Soleus* | - | - | 0.86 | | 0.68 | | 0.80 | 0.67 | 0.79 | | - | | - | - | | - | - | | 0.61 | 0.80 | - | | - |  |
| *Gastrocs* | - | - | - | | 0.88 | | 1.04 | 1.04^a^ | 0.82 | | - | | - | - | | - | - | | 0.80 | 0.88 | - | | - |  |
| *Hamstrings* | 0.76 | 0.84 | 0.97 | | 0.94 | | 0.97 | 0.88 | 0.83 | | 0.95 | | 0.83 | 0.85 | | 0.81 | 0.76 | | 1.01 | 1.11 | 0.84 | | 0.80 |  |
| *Rectus femoris* | 0.80 | 0.80 | - | | 0.90 | | - | 0.90 | - | | 0.80 | | - | - | | - | - | | 0.90 | 0.90 | - | | - |  |
| *Iliopsoas* | - | - | - | | - | | - | - | 1.10 | | 1.35 | | - | - | | 0.94 | - | | 1.19 | 1.22 | 0.94 | | 0.93 |  |
|  |  |  |  | |  | |  |  |  | |  | |  |  | |  |  | |  |  |  | |  |  |
| Strength |  |  |  | |  | |  |  |  | |  | |  |  | |  |  | |  |  |  | |  |  |
| *Hip abductors* | 0.30 | 0.50 | 0.70 | | 0.50 | | 0.70 | 0.70 | 0.50 | | 0.50 | | 0.30 | 0.50 | | 0.30 | 0.30 | | 0.70 | 0.70 | 0.50 | | 0.30 |  |
| *Hip flexors* | 0.50 | 0.50 | 0.70 | | 0.70 | | 0.70 | 0.70 | 0.50 | | 0.70 | | 0.50 | 0.50 | | 0.70 | 0.70 | | 0.70 | 0.70 | 0.50 | | 0.50 |  |
| *Hip extensors* | 0.50 | 0.50 | 0.50 | | 0.50 | | 0.70 | 0.70 | 0.50 | | 0.50 | | 0.30 | 0.30 | | 0.30 | 0.30 | | 0.70 | 0.50 | 0.30 | | 0.30 |  |
| *hip adductors* | 0.50 | 0.50 | 0.70 | | 0.70 | | 0.70 | 0.70 | 0.50 | | 0.70 | | 0.70 | 0.70 | | 0.70 | 0.50 | | 0.70 | 0.70 | 0.50 | | 0.50 |  |
| *Knee flexors* | 0.30 | 0.30 | 0.50 | | 0.50 | | 0.70 | 0.50 | 0.50 | | 0.50 | | 0.30 | 0.50 | | 0.30 | 0.30 | | 0.70 | 0.70 | 0.30 | | 0.30 |  |
| *Knee extensors* | 0.50 | 0.50 | 0.50 | | 0.50 | | 0.70 | 0.50 | 0.50 | | 0.50 | | 0.30 | 0.50 | | 0.70 | 0.50 | | 0.50 | 0.70 | 0.50 | | 0.50 |  |
| *Ankle plantarflexors* | 0.30 | 0.30 | 0.50 | | 0.30 | | 0.50 | 0.30 | 0.20 | | 0.30 | | 0.20 | 0.30 | | 0.20 | 0.20 | | 0.20^b^ | 0.30^b^ | 0.20 | | 0.20 |  |
| *Ankle inversors* | 0.30 | 0.50 | 0.70 | | 0.50 | | 0.70 | 0.50 | 0.20 | | 0.50 | | 0.30 | 0.50 | | 0.30 | 0.20 | | 0.30 | 0.70 | 0.30 | | 0.20 |  |
| *Ankle eversors* | 0.30 | 0.50 | 0.70 | | 0.50 | | 0.70 | 0.70 | 0.20 | | 0.50 | | 0.10 | 0.30 | | 0.30 | 0.20 | | 0.30 | 0.70 | 0.30 | | 0.20 |  |
| *Ankle dorsiflexors* | 0.30 | 0.50 | 0.70 | | 0.50 | | 0.70 | 0.50 | 0.30 | | 0.50 | | 0.20 | 0.50 | | 0.50 | 0.30 | | 0.30 | 0.70 | 0.30 | | 0.20 |  |
| *Abdominal muscles* | 0.30 | | | 0.70 | | 0.70 | | | | 0.50 | | 0.70 | | | 0.30 | | | 0.30 | | | | 0.30 | | |
| *Back muscles* | 0.50 | | | 0.70 | | 0.70 | | | | 0.70 | | 0.70 | | | 0.50 | | | 0.50 | | | | 0.30 | | |

*- the scaling factor is 1*

*^a^ due to same value clinical exam for 0° and 90°*

*^b^ no value recorded during clinical exam, so this is based on the value of the previous clinical exam of this patient*

1. Sensitivity analysis contractures


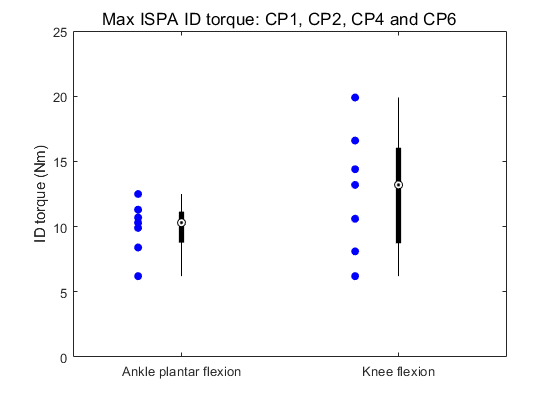
In a subset of the participants and legs (4 participants, 7 legs), the ankle plantarflexion and knee extension torque-angle relationships were measured during an instrumented spasticity assessment [3]. From each participant we selected the trial with the highest measured passive torque at end range of motion for the ankle and knee (Fig. S2).

*Fig. S2: Maximal torque at end range of motion during an instrumented spasticity assessment (IPSA) in four out of the eight subjects (7 legs measured).* *Blue dots correspond to each of the seven assessed legs and the boxplots show the median (black dot), interquartile range (black box), and minimum and maximum (black whiskers).*

The median torque at end range of motion was 10.3 Nm (IQR 2.4 Nm) for the ankle and 13.2 Nm (IQR: 7.4 Nm) for the knee (Table S6). Therefore, we evaluated the sensitivity of the results to using a threshold of 10 Nm instead of 15 Nm in the GENCTR and GEOCTR models (Fig. S3).

**Table S6**

*Boxplot values*

|  | Ankle plantar flexion | Knee flexion |
| --- | --- | --- |
| Median | 10.3 Nm | 13.2 Nm |
| IQR | 2.4 Nm | 7.4 Nm |

*Boxplot values for maximal torque at end of range of motion during an instrumented spasticity assessment (IPSA) in four out of the eight subjects (7 legs measured).*


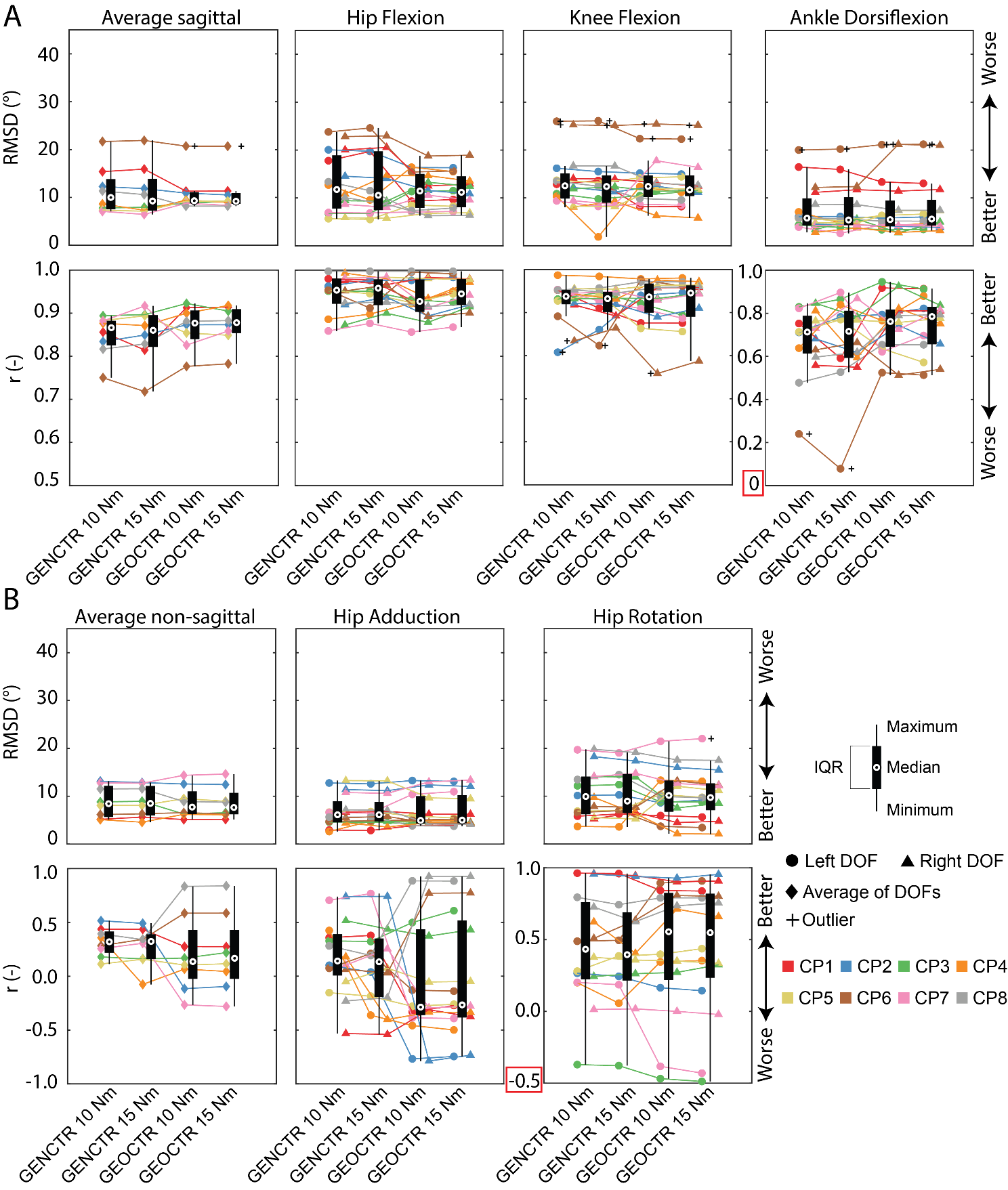


*Fig. S3: Root mean square difference (RMSD) and correlations (r) between simulated and experimental data for thresholds 10 Nm and 15 Nm in both the generic scaled model GEN and the model with bony deformities (GEO).*

1. Moment arm deficits

We modified the weakness scale factor derived from MMT to correct for weakness due to moment arm deficits:

${sf}_{geo}=sf* \left( \frac{T_{max}^{GEO}}{T_{max}^{GEN}} \right)^{-1}$, (s5)

with ${sf}_{geo}$ the corrected weakness scale factor to be used in the model with bony deformities, $sf$ the weakness scale factor derived from MMT, $T_{max}^{GEN}$ the maximal torque the muscle group could generate in the GEN model, and $T_{max}^{GEO}$ the maximal torque the muscle group could generate in the GEO model. The ratio between$T_{max}^{GEO}$ and $T_{max}^{GEN}$ thus reflect the moment arm deficit (Table S8). $T_{max}$ was evaluated with the model in the same position as during MMT (Table S7) and agonistic muscles activated to 100%.

**Table S7**

*Positions in which maximal torque was calculated*

|  | Hip  flexion | Hip adduction | Hip rotation | Knee flexion | Ankle dorsiflexion | Position |
| --- | --- | --- | --- | --- | --- | --- |
| Hip Abductors | 0° | 0° | 0° | 0° | 0° | Side lay |
| Hip Flexors | 90° | 0° | 0° | 90° | 0° | Sit |
| Hip Extensors | 0° | 0° | 0° | 0° | 0° | Prone |
| Hip Adductors | 45° | 0° | 0° | 90° | 0° | Sit |
| Knee Flexors | 0° | 0° | 0° | 0° | 0° | Prone |
| Knee Extensors | 90° | 0° | 0° | 90° | 0° | Sit |
| Ankle Plantar Flexors | 0° | 0° | 0° | 0° | 0° | Stand |
| Ankle Invertors | 90° | 0° | 0° | 90° | 0° | Sit |
| Ankle Evertors | 90° | 0° | 0° | 90° | 0° | Sit |
| Ankle Dorsiflexors | 90° | 0° | 0° | 90° | 0° | Sit |

**Table S8**

*Moment arm deficits*

|  | CP1 | CP2 | CP3 | CP4 | CP5 | CP6 | CP7 | CP8 |
| --- | --- | --- | --- | --- | --- | --- | --- | --- |
| Hip Abductors R | 0.70 | 0.69 | 0.77 | 0.81 | 0.81 | 0.67 | 0.83 | 0.82 |
| Hip Flexors R | 3.07 | 3.73 | 1.46 | 0.90 | 0.96 | 2.35 | 1.33 | 0.92 |
| Hip Extensors R | 0.71 | 0.86 | 0.69 | 0.76 | 0.73 | 0.55 | 0.77 | 0.65 |
| Hip Adductors R | 0.78 | 1.06 | 0.75 | 0.93 | 0.61 | 0.86 | 0.94 | 0.53 |
| Knee Flexors R | 0.84 | 1.42 | 1.54 | 0.92 | 1.02 | 1.17 | 1.40 | 0.92 |
| Knee Extensors R | 1.31 | 1.25 | 1.99 | 0.85 | 1.01 | 1.15 | 1.56 | 0.66 |
| Ankle Plantar Flexors R | 1.09 | 1.04 | 1.07 | 1.13 | 0.85 | 0.79 | 1.22 | 1.16 |
| Ankle Invertors R | 1.15 | 1.06 | 1.09 | 1.17 | 0.95 | 0.82 | 1.10 | 1.12 |
| Ankle Evertors R | 1.12 | 1.04 | 1.08 | 1.14 | 0.99 | 0.85 | 1.04 | 1.09 |
| Ankle Dorsiflexors R | 1.00 | 0.99 | 1.00 | 1.02 | 0.98 | 0.83 | 0.99 | 1.00 |
| Hip abductors L | 0.80 | 0.91 | 0.86 | 0.84 | 0.86 | 0.68 | 0.62 | 0.90 |
| Hip Flexors L | 1.10 | 1.78 | 1.27 | 1.82 | 1.18 | 0.91 | 1.12 | 1.49 |
| Hip Extensors L | 0.72 | 1.13 | 0.69 | 0.42 | 0.70 | 0.68 | 0.92 | 0.72 |
| Hip Adductors L | 0.84 | 0.97 | 0.86 | 0.89 | 0.85 | 0.73 | 0.91 | 0.58 |
| Knee Flexors L | 1.27 | 1.82 | 1.13 | 1.09 | 1.09 | 1.07 | 1.41 | 0.87 |
| Knee Extensors L | 1.34 | 1.39 | 1.17 | 1.10 | 1.02 | 0.96 | 1.22 | 0.49 |
| Ankle Plantar Flexors L | 1.12 | 1.06 | 1.05 | 1.12 | 0.82 | 0.80 | 1.17 | 1.12 |
| Ankle Invertors L | 1.16 | 1.08 | 1.07 | 1.16 | 0.93 | 0.84 | 1.06 | 1.06 |
| Ankle Evertors L | 1.13 | 1.07 | 1.06 | 1.14 | 0.98 | 0.88 | 1.01 | 1.04 |
| Ankle Dorsiflexors L | 1.01 | 0.97 | 1.00 | 1.01 | 0.97 | 0.85 | 0.97 | 0.97 |
| Abdominal muscles | 0.96 | 0.95 | 0.92 | 1.00 | 0.84 | 0.82 | 0.85 | 0.90 |
| Back muscles | 0.99 | 1.00 | 0.94 | 1.03 | 0.87 | 0.85 | 0.87 | 0.92 |

*We estimated moment arm deficits based on the ratio of maximal torque per muscle group (i.e. muscle activation 1) of the GEN and GEO models in the posture assumed during manual muscle testing. Values > 1 represent larger moment arms in GEO, values < 1 represent smaller moment arms in GEO.*

1. Motor control clinical examination data

**Table S9**

*Motor control clinical examination data*

|  | CP1 | | CP2 | | CP3 | | CP4 | | CP5 | | CP6 | | CP7 | | CP8 | | |
| --- | --- | --- | --- | --- | --- | --- | --- | --- | --- | --- | --- | --- | --- | --- | --- | --- | --- |
|  | L | R | L | R | L | R | L | R | L | R | L | R | L | R | L | R |  |
| ***Selectivity total (max = 22)*** | 19,5*^a^* | 21,5*^a^* | 22 | 21,5*^a^* | 22 | 22 | 16,5^b^ | 21*^a^* | 13,5^c^ | 22 | 19*^a^* | 16^b^ | 13,5^c^ | 19*^a^* | 16,5^b^ | 14^c^ |  |
| *Hip flexion* | 2 | 2 | 2 | 2 | 2 | 2 | 2 | 2 | 2 | 2 | 2 | 2 | 1.5 | 2 | 2 | 2 |  |
| *Hip extension* | 2 | 2 | 2 | 2 | 2 | 2 | 2 | 2 | 2 | 2 | 1.5 | 15 | 2 | 2 | 1 | 1.5 |  |
| *Hip abduction* | 2 | 2 | 2 | 2 | 2 | 2 | 2 | 2 | 1.5 | 2 | 1.5 | 15 | 1.5 | 2 | 2 | 2 |  |
| *Hip adduction* | 2 | 2 | 2 | 2 | 2 | 2 | 2 | 2 | 2 | 2 | 2 | 2 | 2 | 1.5 | 2 | 2 |  |
| *Knee flexion* | 1 | 1.5 | 2 | 2 | 2 | 2 | 2 | 1.5 | 1.5 | 2 | 1.5 | 1.5 | 1.5 | 2 | 1.5 | 2 |  |
| *Knee extension* | 2 | 2 | 2 | 2 | 2 | 2 | 1.5 | 2 | 1.5 | 2 | 1.5 | 1.5 | 1.5 | 1.5 | 1.5 | 1.5 |  |
| *Ankle dorsiflexion knee 90°* | 2 | 2 | 2 | 2 | 2 | 2 | 1.5 | 2 | 0.5 | 2 | 2 | 1.5 | 0.5 | 2 | 1 | 0.5 |  |
| *Ankle dorsiflexion knee 0°* | 1.5 | 2 | 2 | 1.5 | 2 | 2 | 1.5 | 2 | 0.5 | 2 | 2 | 1.5 | 0.5 | 2 | 1 | 0.5 |  |
| *Ankle plantar flexion* | 2 | 2 | 2 | 2 | 2 | 2 | 1 | 2 | 1 | 2 | 2 | 2 | / | / | 1.5 | 1 |  |
| *Ankle inversion* | 2 | 2 | 2 | 2 | 2 | 2 | 0.5 | 2 | 1 | 2 | 1.5 | 0.5 | 1 | 2 | 1.5 | 0.5 |  |
| *Ankle eversion* | 1 | 2 | 2 | 2 | 2 | 2 | 0.5 | 1.5 | / | 2 | 1.5 | 0.5 | 1.5 | 2 | 1.5 | 0.5 |  |
|  |  |  |  |  |  |  |  |  |  |  |  |  |  |  |  |  |  |
| ***SCALE total (max = 10)*** | 8*^a^* | 9*^a^* | 10 | 10 | 10 | 10 | 6^b^ | 10 | 6^b^ | 9*^a^* | 7^b^ | 6^b^ | / | / | 9*^a^* | 7^b^ |  |
| *Hip* | 2 | 2 | 2 | 2 | 2 | 2 | 2 | 2 | 2 | 2 | 2 | 2 | / | / | 2 | 2 |  |
| *Knee* | 2 | 2 | 2 | 2 | 2 | 2 | 2 | 2 | 2 | 2 | 2 | 2 | / | / | 2 | 2 |  |
| *Ankle* | 2 | 2 | 2 | 2 | 2 | 2 | 2 | 2 | 1 | 2 | 2 | 1 | / | / | 2 | 1 |  |
| *Subtalar joint* | 1 | 2 | 2 | 2 | 2 | 2 | 0 | 2 | 1 | 2 | 1 | 1 | / | / | 2 | 1 |  |
| *Toes* | 1 | 1 | 2 | 2 | 2 | 2 | 0 | 2 | 0 | 1 | 0 | 0 | / | / | 1 | 1 |  |
|  |  |  |  |  |  |  |  |  |  |  |  |  |  |  |  |  |  |
| ***Spasticity total (min = 0)*** | 5^b^ | 3^b^ | 3^b^ | 9^c^ | 1*^a^* | 4^b^ | 7,5^c^ | 6^b^ | 2*^a^* | 4^b^ | 2*^a^* | 2*^a^* | 11,5^c^ | 6^b^ | 7^c^ | 8^c^ |  |
| *Hip flexors* | 0 | 0 | 0 | 1 | 0 | 0 | 1 | 0 | 0 | 1 | 0 | 0 | 0 | 0 | 1.5 | 1.5 |  |
| *Hip adductors knee 0°* | 1 | 0 | 0 | 1 | 0 | 0 | 0 | 0 | 0 | 0 | 0 | 0 | 1 | 0 | 1 | 1.5 |  |
| *Hip adductors knee 90°* | 0 | 0 | 0 | 0 | 0 | 0 | 0 | 0 | 0 | 0 | 0 | 0 | 1 | 0 | 0 | 0 |  |
| *Hamstrings* | 2 | 1 | 1 | 1.5 | 0 | 1 | 3 | 1.5 | 1 | 1 | 1 | 1 | 1.5 | 1 | 2 | 2 |  |
| *Duncan Ely* | 1 | 1 | 0 | 1 | 0 | 0 | 0 | 1.5 | 0 | 1 | 0 | 0 | 1 | 0 | 0 | 1 |  |
| *Soleus* | 0 | 0 | 1 | 1.5 | 0 | 1.5 | 1.5 | 1.5 | 0 | 0 | 0 | 0 | 2 | 1.5 | 1 | 1 |  |
| *Gastrocnemii* | 1 | 1 | 1 | 2 | 1 | 1.5 | 2 | 1.5 | 1 | 1 | 1 | 1 | 3 | 2 | 1.5 | 1 |  |
| *Tibialis Posterior* | 0 | 0 | 0 | 1 | 0 | 0 | 0 | 0 | 0 | 0 | 0 | 0 | 2 | 1.5 | 0 | 0 |  |

*Measured values were categorized with ^a^ slight impairment, ^b^ considerable impairment and ^c^ severe impairment. No categorization means the value is within normal range. For selectivity and SCALE a higher score is closer to normal, for spasticity a lower score is closer to normal.*

*/ no values recorded during the clinical examination.*

1. Shapley values

To disentangle the relative impact of multiple interacting impairments, we employed Shapley values, a method originating from cooperative game theory [4]. Classically, Shapley values quantify each player’s fair contribution to the overall outcome of a game, taking interaction effects into account. Here, we used Shapley values to quantify each impairment’s contribution to the RMSD or correlation between simulations and experimental data. We evaluate how much each impairment contributed to the RMSD or correlation by considering all possible subsets of impairments (i.e. our eight models) and computing the marginal gain of including a given impairment. The Shapley value then averages these marginal contributions across all subset combinations, weighted to ensure a fair allocation.

The added value of using Shapley values is that they quantify the marginal contribution of each impairment across all simulations, rather than just comparing simulations based on two models directly. In other words, instead of asking “what happens if we compare a model with weakness to one without weakness”, Shapley values ask “on average, how much does weakness change performance when introduced into every possible context of other impairments?” Hence, the results may look different from a simple comparison like GEN vs. GENWEAK. The GEN vs. GENWEAK comparison only reflects the effect of weakness in those two specific configurations, while the Shapley value accounts for the role of weakness across all model variations. Therefore, it gives a more comprehensive overview of the contribution of the different impairments to the altered gait.

The Shapley value $\varphi_{i}$for each impairment $i$ is given by:

$\varphi_{i} =\sum_{\begin{aligned} S \subseteq N \\ i \notin S \end{aligned}} \frac{\left| S \right|!\left( n-\left| S \right|-1 \right)!}{n!} \left( v\left( S\cup\left\{ i \right\} \right)-v\left( S \right) \right)$ , (s6)

where $N$ is the set of all impairments, $S$ is a subset of $N$ not containing $i$, $v\left( S \right)$ is the performance metric (RMSD or correlation) for subset $S$, $\left| S \right|$ is the number of other impairments in $S$, and $n$ is the total number of impairments.

Furthermore, interaction values $I_{ij}$ are calculated between impairments $i$ and $j$ estimating the contribution of that subset of modeled impairments.

$I_{ij} =\sum_{\begin{aligned} S \subseteq N \\ i,j \notin S \end{aligned}} \frac{\left| S \right|!\left( n-\left| S \right|-2 \right)!}{2\left( n-1 \right)!} \left( v\left( S\cup\left\{ i,j \right\} \right)- v\left( S\cup\left\{ i \right\} \right)- v\left( S\cup\left\{ j \right\} \right)+v\left( S \right) \right)$ , (s7)

In this formula $N$ is the complete set of impairments, $S$ is any group of impairments that does not include $i$ or $j$, $v\left( S \right)$ is the performance score (RMSD or correlation) for that group; $\left| S \right|$ is how many impairments are in the group; and $n$ is the total number of impairments overall.

To further illustrate how Shapley values are computed, consider the following examples.

There are three impairments (weakness, contractures, bony deformities), which results in eight possible combinations of these impairments, including the case with no impairments. For each impairment, there are four models containing that impairment and four models without it.

For each model that includes the impairment $i$ (denoted as $S\cup\left\{ i \right\}$), we subtract the RMSD of the corresponding model without that impairment ($S$).

For weakness, this results in the following differences:

- RMSD(GENWEAK) − RMSD(GEN)
- RMSD(GENFULL) − RMSD(GENCTR)
- RMSD(GEOWEAK) − RMSD(GEO)
- RMSD(GEOFULL) − RMSD(GEOCTR)

This difference corresponds to the factor $\left( v\left( S\cup\left\{ i \right\} \right)-v\left( S \right) \right)$ in equation s6, and is often referred to as the marginal gain.

That factor is weighted by:

$$\frac{\left| S \right|!\left( n-\left| S \right|-1 \right)!}{n!}$$

where $\left| S \right|$ is the number of other impairments in $S$ (e.g. for GEOCTR it is 2, for GEN it is 0) and $n$ is the total number of impairments (in our case 3). The possible weights therefore are:

- 0.3333 when $v\left( S \right)$ contains no impairments (e.g., GEN)
- 0.1666 when $v\left( S \right)$ contains one other impairment
- 0.3333 when $v\left( S \right)$ contains two other impairments

Thus, greater importance is given to the performance when either none or all other impairments are included.

The Shapley values were calculated per subject. Later, boxplots were calculated with those values to make more general interpretations.

Modeling contractures results in a clear median decrease in RMSD in the sagittal plane while modeling weakness results in a median increase and modeling bony deformities results in no difference (Fig. 3). When modeling both contractures and either weakness or bony deformities, there is an additional (on top of the sum of the individual effects) decrease in median RMSD. This is not the case for modeling both weakness and bony deformities, which results in an increase in median RMSD. In the non-sagittal plane, modeling all individual impairments results in an increase in RMSD. Modeling weakness in combination with either contractures or bony deformities decreases RMSD, while modeling contractures and bony deformities increases RMSD.

In the sagittal plane, Shapley values for the correlation are around zero, indication a negligible effect of both individual and multiple impairments (Fig. 3). In contrast, in the non-sagittal plane Shapley values vary widely although also with a median effect on the correlation close to zero. But, interactions between bony deformities and either weakness or contractures improve the correlation.

1. SPM1D analysis

Outcomes of the SPM1D analysis of differences in how well different models capture the experimental gait kinematics (difference between simulated and experimental gait kinematics) are reported in Fig. S4 and Fig. S5. We considered this analysis exploratory because our study design led to many different models.


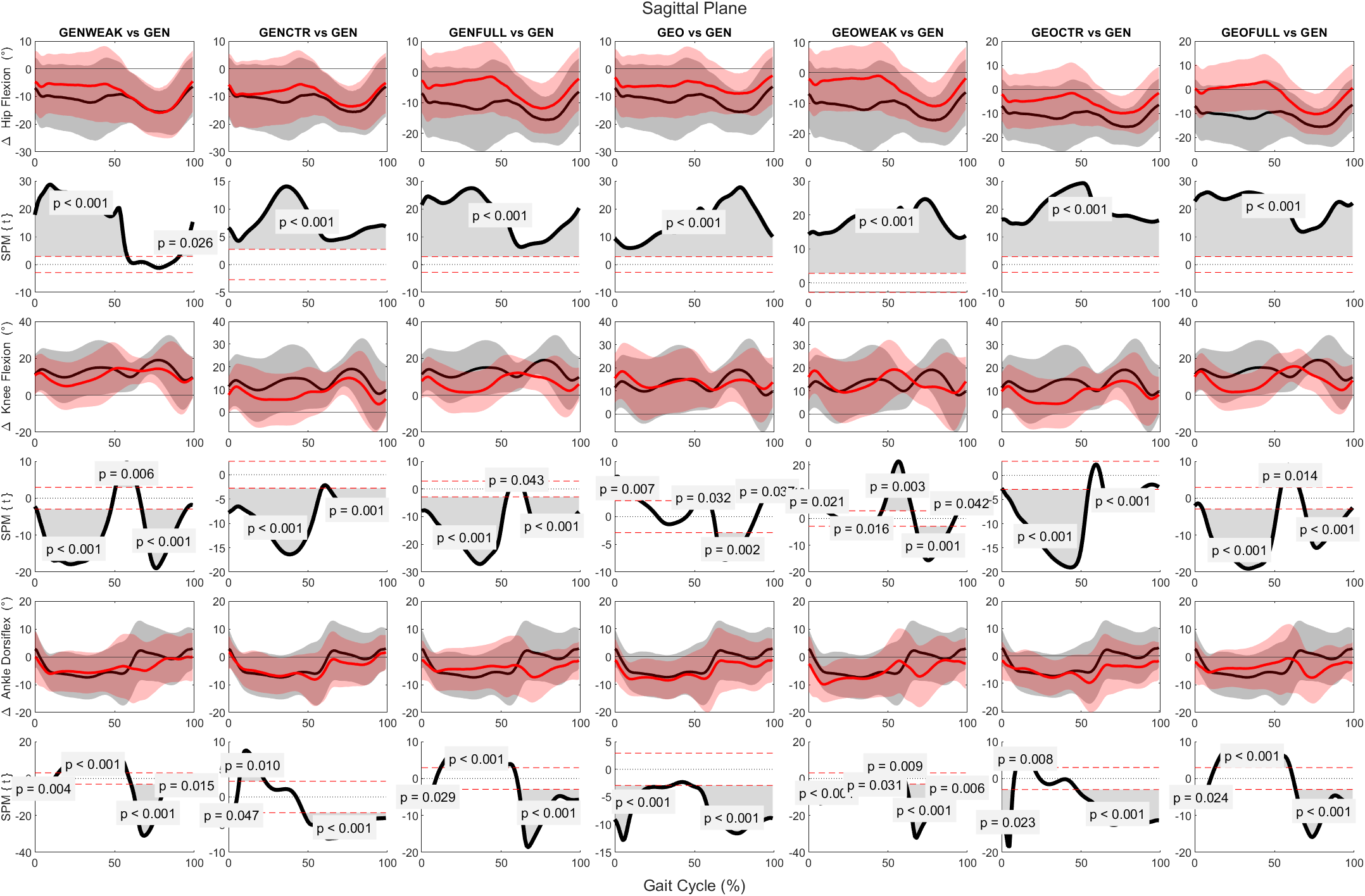


*Fig. S4: Results from SPM1D analysis testing differences in the agreement between simulated and experimental joint kinematics (Δ) between each of the personalized models (columns) and the generic model in* ***the sagittal plane****. Right and left degrees of freedom were considered together. Differences closer to zero correspond to a better agreement between simulated and experimental kinematics. Black line and grey shaded area in the Δ-plots are mean ± SD for GEN. Red line and red shaded area in the Δ-plots are mean ± SD for model with impairment(s). SPM{t} indicates the statistical parametric map of t-values with two-tailed inference set at an alpha level of 0.05. p-values reflect if a set of t-values is significant indicating a difference between the two signals.*

*
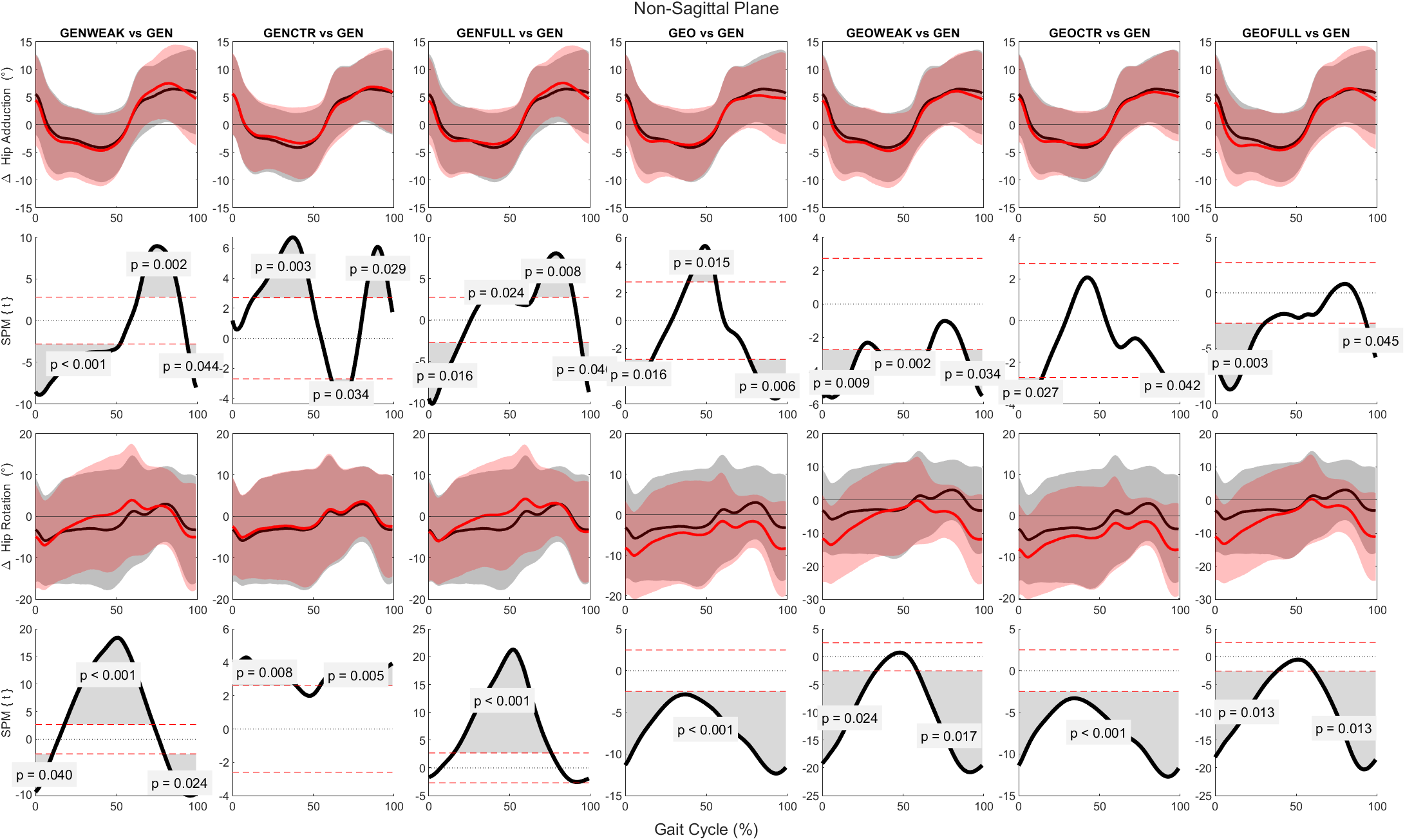
*

*Fig S5: Results from SPM1D analysis testing differences in the agreement between simulated and experimental joint kinematics (Δ) between each of the personalized models (columns) and the generic model in* ***the non-sagittal plane****. Right and left degrees of freedom were considered together. Differences closer to zero correspond to a better agreement between simulated and experimental kinematics. Black line and grey shaded area in the Δ-plots are mean ± SD for GEN. Red line and red shaded area in the Δ-plots are mean ± SD for model with impairment(s). SPM{t} indicates the statistical parametric map of t-values with two-tailed inference set at an alpha level of 0.05. p-values reflect if a set of t-values is significant indicating a difference between the two signals.*

1. Simulated and experimental kinematics per subject


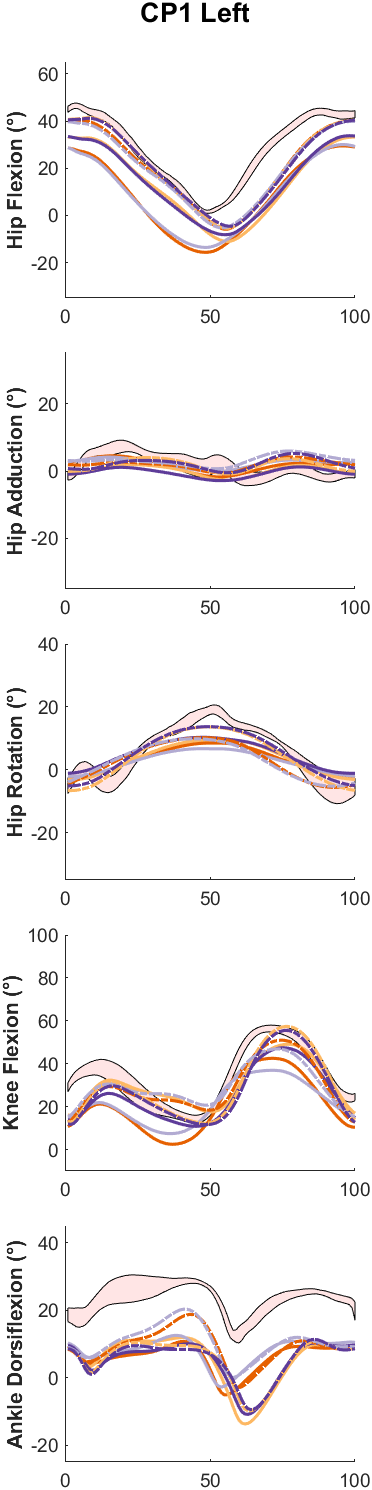

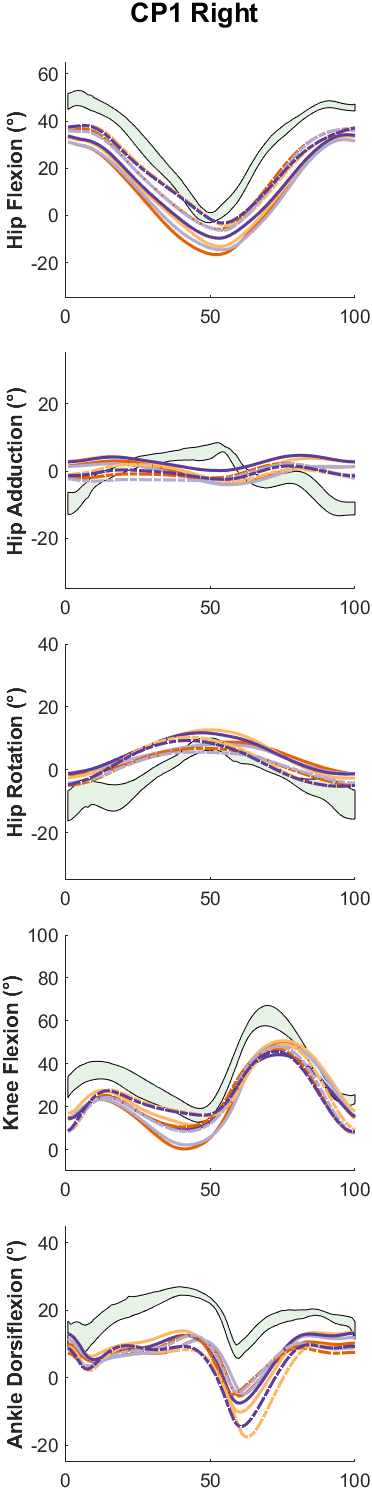

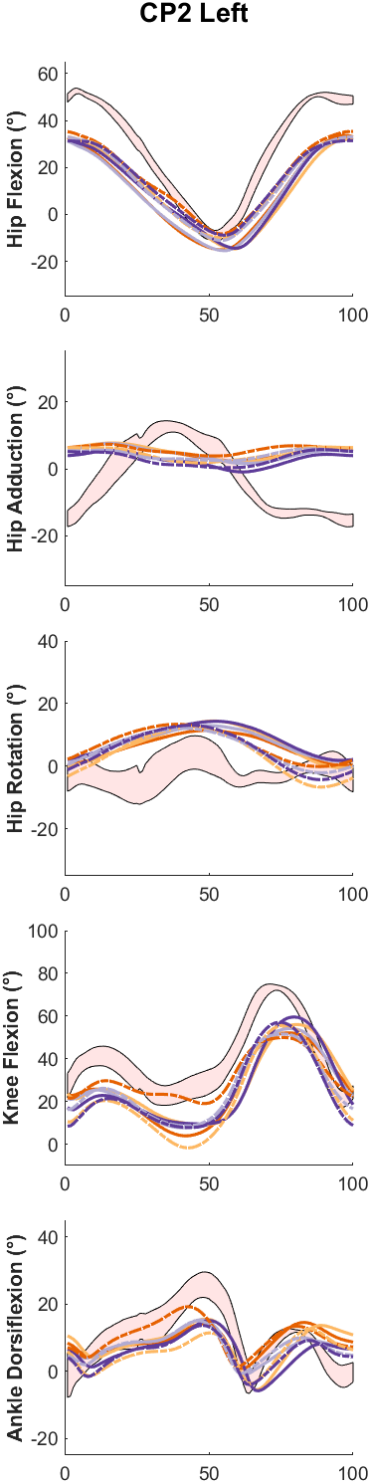

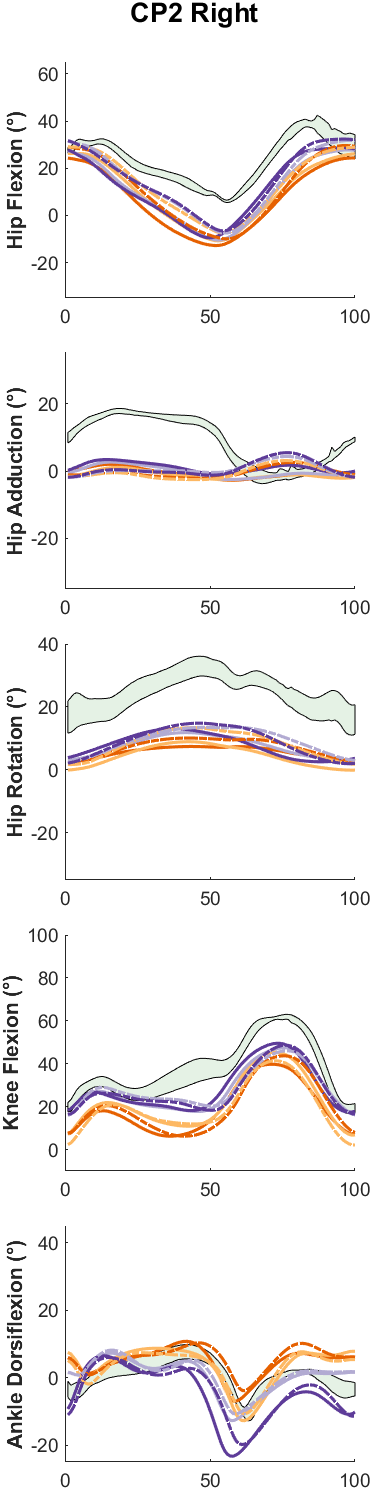


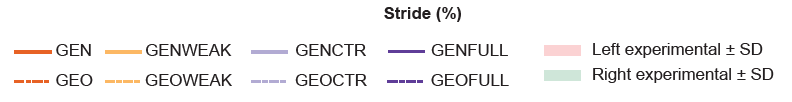


*Fig. S6:* *Experimental and simulated kinematics of CP1 and CP2. GEN is the generic scaled model, GENWEAK is GEN with weakness, GENCTR is GEN with contractures, GENFULL is GEN with both weakness and contractures. GEO is the model with MRI-based deformities, GEOWEAK is GEO with weakness, GEOCTR is GEO with contractures, and GEOFULL is GEO with both weakness and contractures. GEN represents how a typically developing individual with the same dimensions as the patient would walk.*


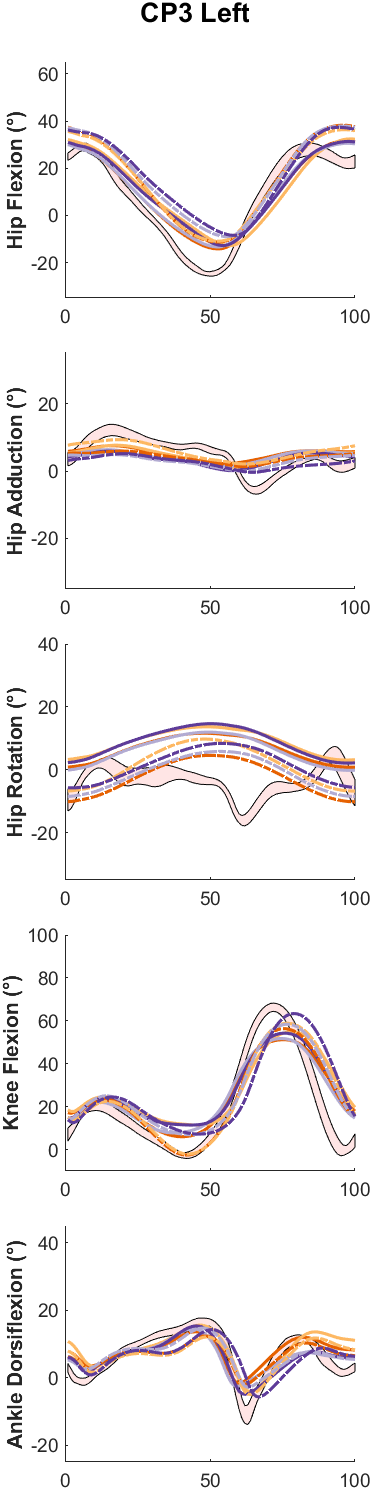

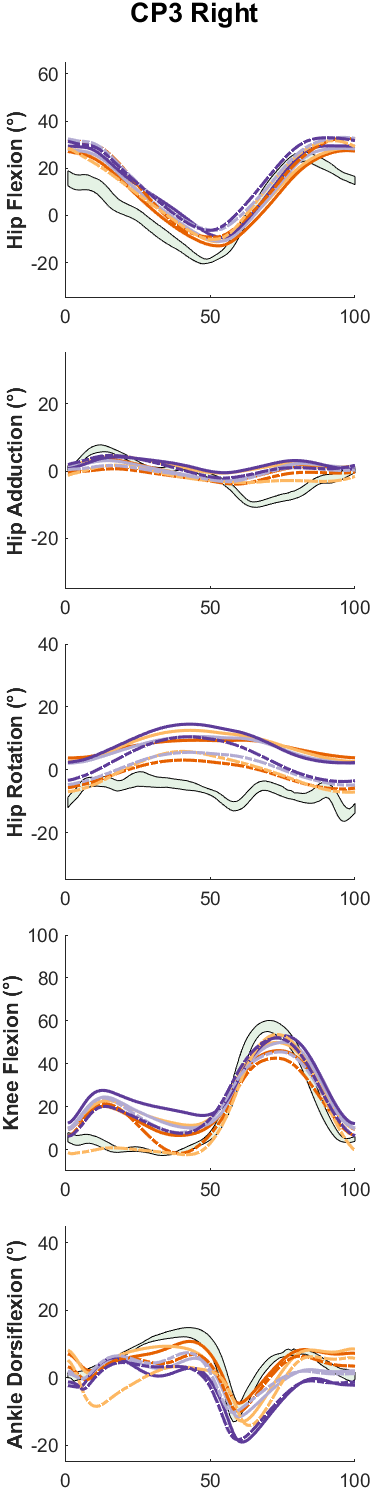

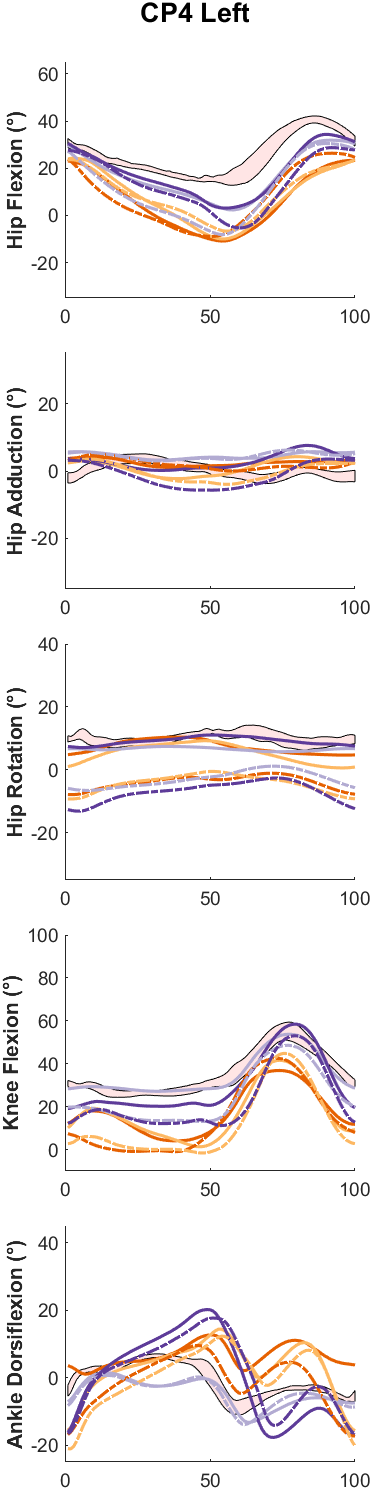

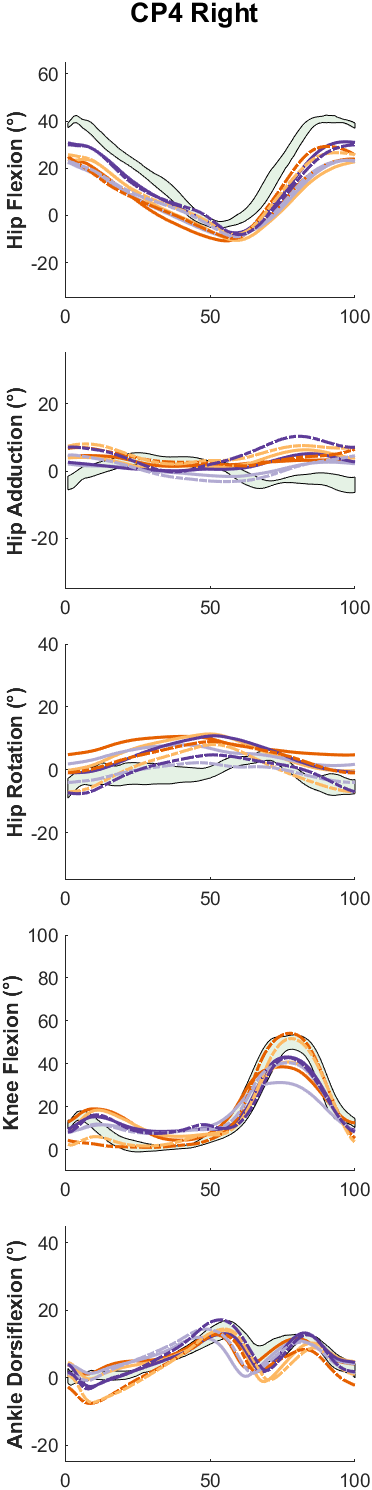


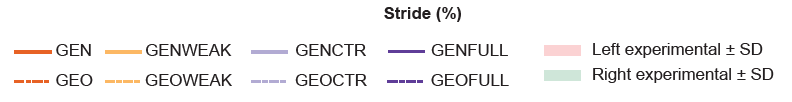


*Fig. S7:* *Experimental and simulated kinematics of CP3 and CP4. GEN is the generic scaled model, GENWEAK is GEN with weakness, GENCTR is GEN with contractures, GENFULL is GEN with both weakness and contractures. GEO is the model with MRI-based deformities, GEOWEAK is GEO with weakness, GEOCTR is GEO with contractures, and GEOFULL is GEO with both weakness and contractures. GEN represents how a typically developing individual with the same dimensions as the patient would walk.*


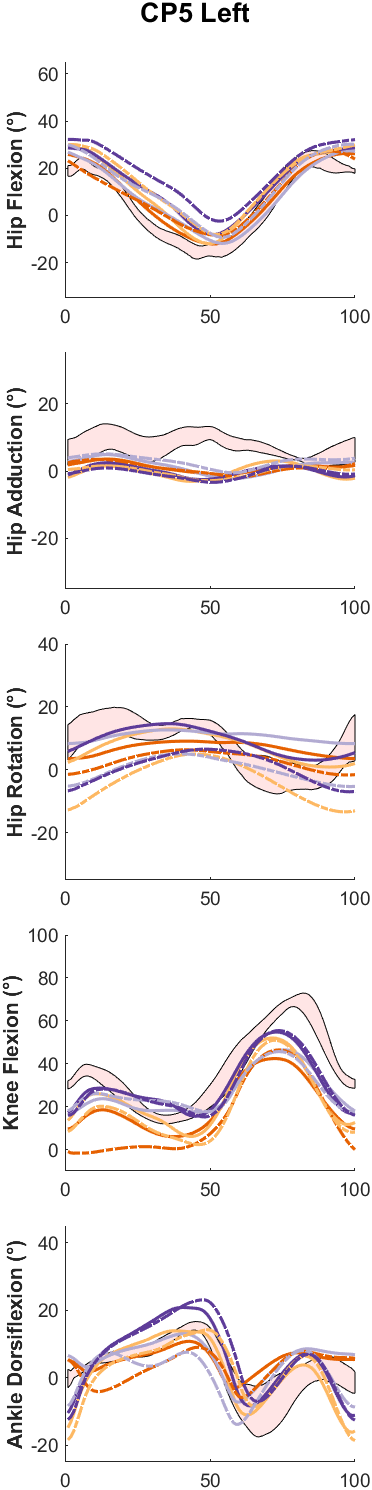

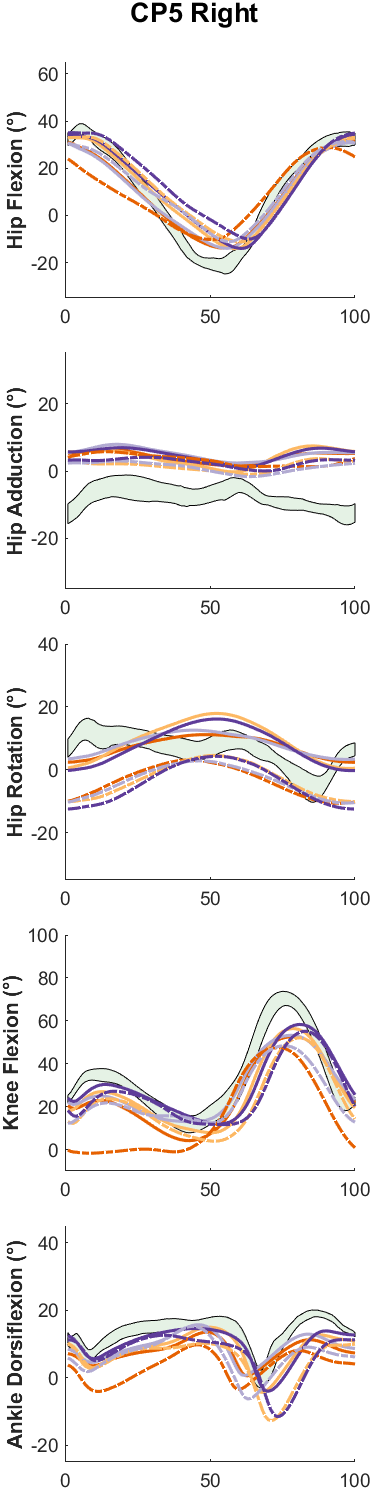

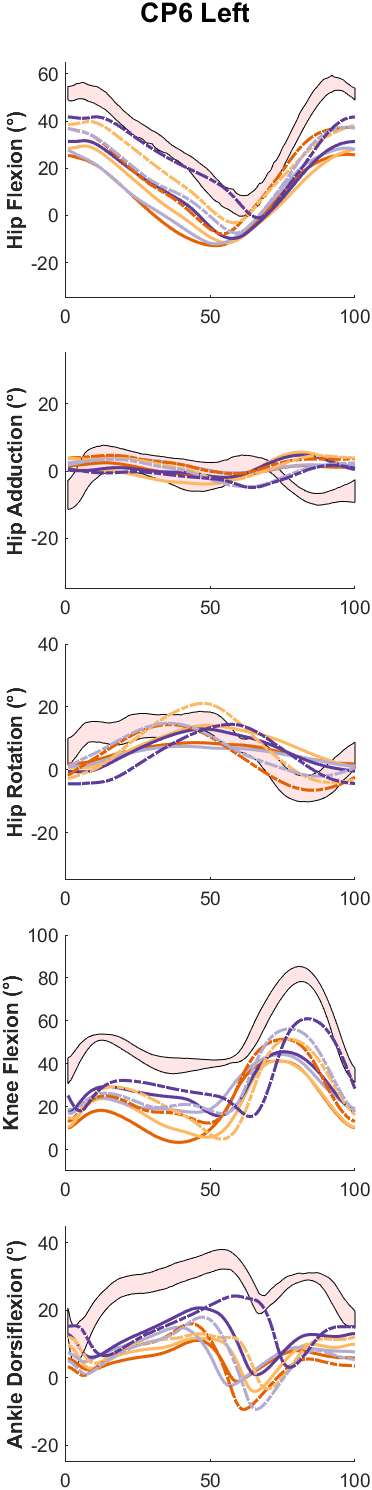

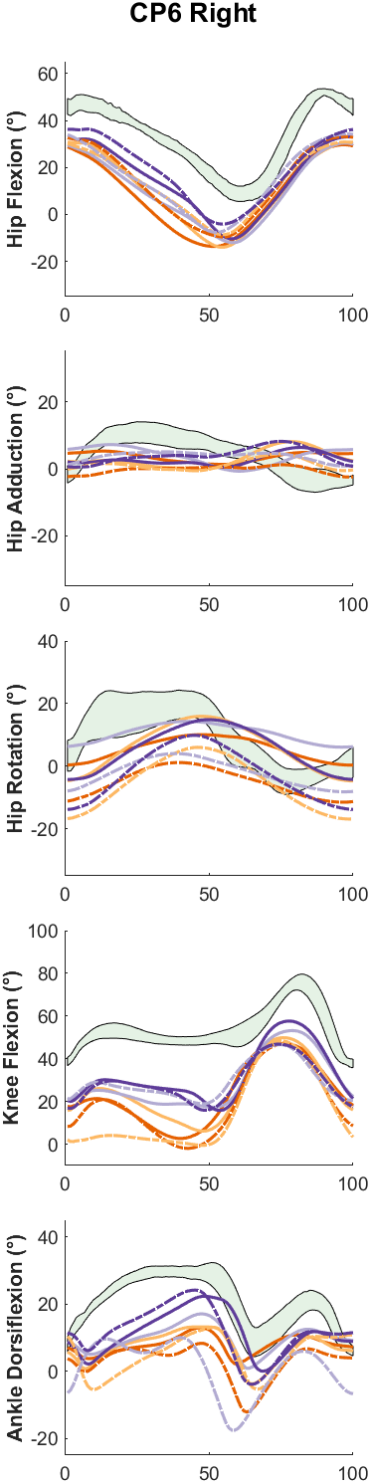


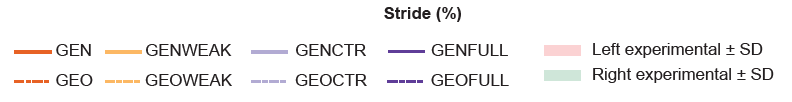


*Fig. S8:* *Experimental and simulated kinematics of CP5 and CP6. GEN is the generic scaled model, GENWEAK is GEN with weakness, GENCTR is GEN with contractures, GENFULL is GEN with both weakness and contractures. GEO is the model with MRI-based deformities, GEOWEAK is GEO with weakness, GEOCTR is GEO with contractures, and GEOFULL is GEO with both weakness and contractures. GEN represents how a typically developing individual with the same dimensions as the patient would walk.*


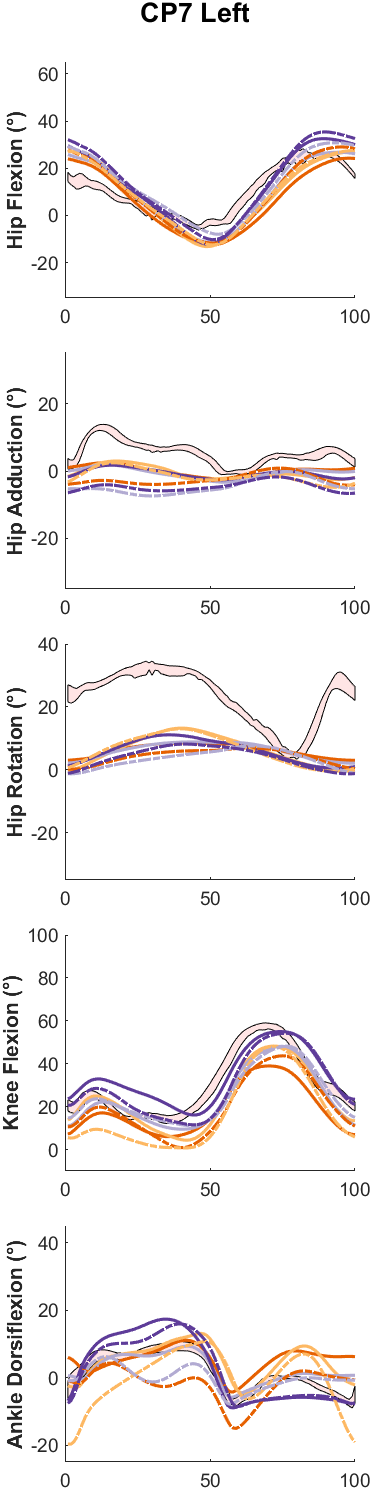

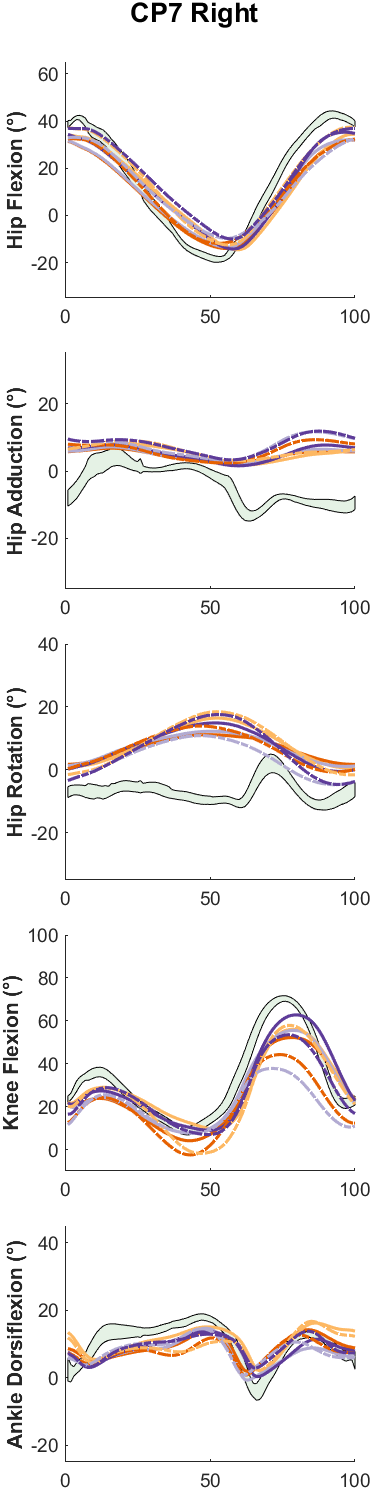

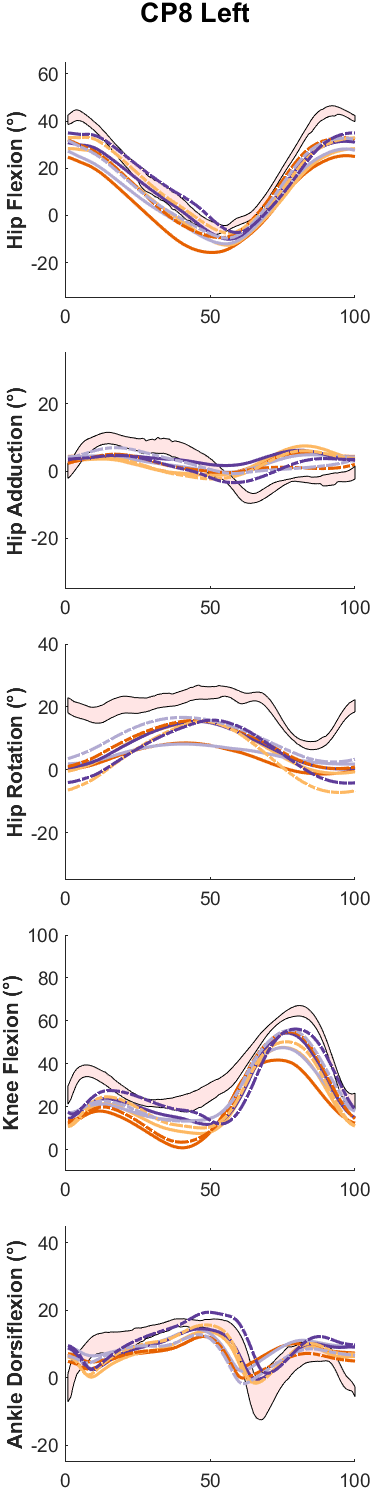

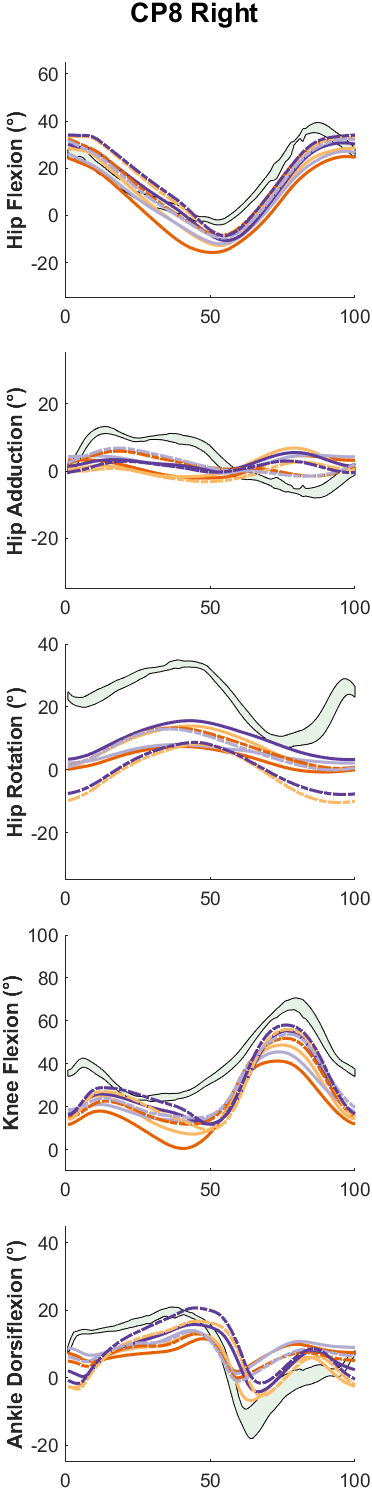


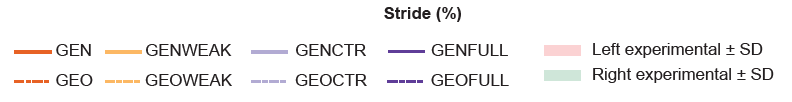


*Fig. S9:* *Experimental and simulated kinematics of CP7 and CP8. GEN is the generic scaled model, GENWEAK is GEN with weakness, GENCTR is GEN with contractures, GENFULL is GEN with both weakness and contractures. GEO is the model with MRI-based deformities, GEOWEAK is GEO with weakness, GEOCTR is GEO with contractures, and GEOFULL is GEO with both weakness and contractures. GEN represents how a typically developing individual with the same dimensions as the patient would walk.*

1. RMSD and CC differences between models, per subject

*Fig. S10: Difference in RMSD between simulations and experimental data between personalized models and the reference model GEN for all patients and studied degrees of freedom on the right side. A green bar indicates an assumed contribution of the modeled impairment to altered gait. A red bar indicates modeled impairments are assumed to not contribute to the observed gait pattern. GEN is the generic scaled model, GENWEAK is GEN with weakness, GENCTR is GEN with contractures, GENFULL is GEN with both weakness and contractures. GEO is the model with MRI-based deformities, GEOWEAK is GEO with weakness, GEOCTR is GEO with contractures, and GEOFULL is GEO with both weakness and contractures. GEN represents how a typically developing individual with the same dimensions as the patient would walk.*

*Fig. S11: Difference in RMSD between simulations and experimental data between personalized models and the reference model GEN for all patients and studied degrees of freedom on the left side. A green bar indicates an assumed contribution of the modeled impairment to altered gait. A red bar indicates modeled impairments are assumed to not contribute to the observed gait pattern. GEN is the generic scaled model, GENWEAK is GEN with weakness, GENCTR is GEN with contractures, GENFULL is GEN with both weakness and contractures. GEO is the model with MRI-based deformities, GEOWEAK is GEO with weakness, GEOCTR is GEO with contractures, and GEOFULL is GEO with both weakness and contractures. GEN represents how a typically developing individual with the same dimensions as the patient would walk.*

*Fig. S12: Difference in r between personalized models and the reference model GEN for all patients and studied degrees of freedom on the right. A green bar indicates an assumed contribution of the modeled impairment to altered gait. A red bar indicates modeled impairments are assumed to not contribute to the observed gait pattern. GEN is the generic scaled model, GENWEAK is GEN with weakness, GENCTR is GEN with contractures, GENFULL is GEN with both weakness and contractures. GEO is the model with MRI-based deformities, GEOWEAK is GEO with weakness, GEOCTR is GEO with contractures, and GEOFULL is GEO with both weakness and contractures. GEN represents how a typically developing individual with the same dimensions as the patient would walk.*

*Fig. S13: Difference in r between personalized models and the reference model GEN for all patients and studied degrees of freedom on the left. A green bar indicates an assumed contribution of the modeled impairment to altered gait. A red bar indicates modeled impairments are assumed to not contribute to the observed gait pattern. GEN is the generic scaled model, GENWEAK is GEN with weakness, GENCTR is GEN with contractures, GENFULL is GEN with both weakness and contractures. GEO is the model with MRI-based deformities, GEOWEAK is GEO with weakness, GEOCTR is GEO with contractures, and GEOFULL is GEO with both weakness and contractures. GEN represents how a typically developing individual with the same dimensions as the patient would walk.*

1. References

[1] M. Goudriaan, A. Nieuwenhuys, S.-H. Schless, N. Goemans, G. Molenaers, and K. Desloovere, “A new strength assessment to evaluate the association between muscle weakness and gait pathology in children with cerebral palsy,” *PLOS ONE*, vol. 13, no. 1, Art. no. 1, Jan. 2018, doi: 10.1371/journal.pone.0191097.

[2] I. Vandekerckhove *et al.*, “Anthropometric-related percentile curves for muscle size and strength of lower limb muscles of typically developing children,” *J. Anat.*, vol. 247, no. 2, pp. 348–362, 2025, doi: 10.1111/joa.14241.

[3] L. Bar-On *et al.*, “A clinical measurement to quantify spasticity in children with cerebral palsy by integration of multidimensional signals,” *Gait Posture*, vol. 38, no. 1, pp. 141–147, May 2013, doi: 10.1016/j.gaitpost.2012.11.003.

[4] L. S. Shapley, “A value for n-person games,” 1953, Accessed: Aug. 26, 2025. [Online]. Available: https://www.torrossa.com/gs/resourceProxy?an=5641636&publisher=FZO137#page=87
